# Cytokine expression in *Treponema pallidum* infection

**DOI:** 10.1186/s12967-019-1947-7

**Published:** 2019-06-11

**Authors:** N. Kojima, J. C. Siebert, H. Maecker, Y. Rosenberg-Hasson, S. R. Leon, S. K. Vargas, K. A. Konda, C. F. Caceres, J. D. Klausner

**Affiliations:** 1 David Geffen School of Medicine, University of California Los Angeles, 10833 Le Conte Ave, Los Angeles, CA 90095 USA; 2 CytoAnalytics, Denver, CO 80113 USA; 3 Human Immune Monitoring Center, Institute for Immunity, Transplantation, and Infection, Stanford University School of Medicine, Stanford, 94305 USA; 4 Laboratory of Sexual Health and Unit of Health, Sexuality and Human Development, Universidad Peruana Cayetano Heredia, Lima, USA; 5 Fielding School of Public Health, University of California Los Angeles, Los Angeles, 90024 USA

**Keywords:** Cytokine, Cytokines, Syphilis, *Treponema pallidum*

## Abstract

**Background:**

Current syphilis tests cannot distinguish between active and past syphilis among patients with serofast rapid plasma reagin (RPR) titers. We investigated whether cytokine profiles might provide insight in the differentiation of active and treated syphilis.

**Methods:**

We collected quarterly serum samples from participants at risk for incident syphilis in a prospective cohort study of men and male-to-female transgender women. We defined incident syphilis as a new RPR titer ≥ 1:8 or a fourfold increase from a prior RPR titer and a positive *Treponema pallidum* particle agglutination assay. We measured cytokine expression using a 63-multiplex bead-based Luminex assay (eBiosciences/Affymetrix, San Diego, California, USA). We used tertile bins and Chi square tests to identify differences in proportions of cytokines between samples from patients with active and treated syphilis. We constructed a network of cytokine profiles from those findings. We used R software (R version 3.4.1, R, Vienna, Austria) to fit models.

**Results:**

We identified 20 pairs of cytokines (out of 1953 possible pairs) that differed between active and treated syphilis. From those, we identified three cytokine networks of interest: an Eotaxin–Rantes–Leptin network, a Mig-IL1ra-Trail-CD40L network, and an IL12p40-IL12p70 network.

**Conclusions:**

Differences in cytokine profiles are present among men and male-to-female transgender women with active and treated syphilis. Cytokine assays may be a potentially useful tool for identifying active syphilis among patients with serologic syphilis reactivity.

**Electronic supplementary material:**

The online version of this article (10.1186/s12967-019-1947-7) contains supplementary material, which is available to authorized users.

## Background

Syphilis, caused by *Treponema pallidum pallidum*, is an identifiable and curable infection [1, 2]. The diagnosis of syphilis mainly relies on serological testing for reactivity to both non-treponemal (cardiolipin) and treponemal antigens [3]. Treponemal assays are primarily used to confirm the presence of past or current syphilis, whereas non-treponemal assays are mostly used to monitor the “activity” of infection [4]. Direct detection methods like dark-field microscopy, direct fluorescent antibody testing, nucleic acid amplification testing, and examination of cerebrospinal fluid can also be used, however they are often insensitive and difficult to access [5].

There are limitations to serologic testing for syphilis. Depending on the stage of infection and severity of immunosuppression [6], serologic testing has varying degrees of sensitivity and specificity [4]. It is estimated that commonly used treponemal and non-treponemal tests have a sensitivity ranging from 76 to 100% and specificity ranging from 97 to 99% when compared to direct detection during symptomatic syphilis [7]. Additionally, the optimal clinical management of the 15% to 41% of persons who remain serofast following treatment for syphilis can be unclear [8]. The serofast phenomenon occurs because non-treponemal assays detect host antibodies (cardiolipin), which can have long half-lives [8, 9]. False-positive non-treponemal tests can also occur among those with febrile illnesses, immunizations, hepatitis C virus infection, connective tissue disease, intravenous drug use, malignancy, older age, malaria, Chagas disease, tuberculosis, and leprosy [7, 10, 11]. The antibodies that treponemal assays detect can remain present for life, even after successful treatment for syphilis. False-positive test results can also be due to due to other spirochete infections (*Borrelia* spp.) and from commensal microorganisms [7, 12]. To improve upon the limitations of serologic testing, diagnostic algorithms have been created to increase testing sensitivity and specificity [4]. Those algorithms are based on serologic testing with a non-treponemal assay with a verification treponemal assay. However, those algorithms can miss previously treated, early untreated, and late latent cases of syphilis due to non-reactive non-treponemal assays. Current diagnostic algorithms are unable to distinguish currently active from previously treated syphilis.

Studies using specific inflammatory cytokines as potential markers of infection resolution among patients with primary and secondary syphilis have found promising results [13, 14]. However, those studies tested few cytokines and did not have measurements of cytokine concentrations prior to incident syphilis. We investigated whether cytokine profiles might provide insight into the “activity” of syphilis by measuring 63 plasma cytokines in a longitudinal cohort of participants with no syphilis, incident syphilis, and treated syphilis. We aimed to address three questions: [1] are changes in cytokine concentrations associated with changes in rapid plasma reagin (RPR) titers; [2] does cytokine response depend on directionality of changes in RPR titers, i.e., increasing versus decreasing RPR titers; and [3] can observing one or more cytokines better distinguish participants with active infection and treated syphilis.

## Methods

From 2013 to 2015, a cohort of men who have sex with men (MSM) and male-to-female transwomen (transwomen) who engaged in behavior that put them at risk for syphilis were recruited and enrolled into an observational study in Lima, Peru [15, 16]. Briefly, participants attended a sexually transmitted infection clinic every 3 months for a period of 2 years. During study visits, participants were interviewed, received routine syphilis and human immunodeficiency virus (HIV) infection testing, and had clinical specimens collected.

### Collection and storage of blood serum

Whole venous blood samples were collected from participants every 3 months. Sampled blood was held at room temperature for clot formation and then underwent centrifugation. Sera from samples were frozen and stored at − 80 °C at the Universidad Peruana Cayetano Heredia Sexual Health Laboratory.

### Syphilis testing among participants

Participants were tested for syphilis with serum RPR tests (BD Macro-Vue™ RPR Card Test Kit, Beckton Dickinson, Franklin Lakes, NJ) and *Treponema pallidum* particle agglutination (TPPA) tests (Serodia, Fujirebio Diagnostics Inc., Tokyo, Japan), using a cutoff value of ≥ 1:80. Participants were also tested for HIV infection (Alere Determine™ HIV 1/2, Alere Inc., Waltham, MA, USA and NEW LAV BLOT I, Bio-Rad, France).

### Selection of samples for cytokine analysis

Incident syphilis was defined as an RPR titer that was ≥ 1:8 or a fourfold increase from the prior RPR titer, and a positive TPPA test. Treated syphilis was defined as a fourfold or larger reduction in RPR titer following syphilis treatment. We selected a serum sample collected during a clinic visit in which a participant was found to have incident syphilis, in addition to a serum sample collected from the same participant when they did not have syphilis from a temporally adjacent clinic visit. Samples collected from participants with incident syphilis were coded as “infection-yes” and samples collected from participants without syphilis were coded as “infection-no.” Additionally, samples were stratified by RPR titers, as well as whether they were collected prior to incident syphilis, i.e., “no syphilis,” and after the participant was treated for syphilis, i.e., “cured”. All samples were shipped on frozen carbon dioxide to the Human Immune Monitoring Center at Stanford University for analysis.

### Luminex assay and magnetic bead kits

The simultaneous measurement of 63 cytokines (eBiosciences/Affymetrix, San Diego, California, USA) in serum samples was performed using a multiplex bead-based Luminex assay. See Additional file 1: Table S1 for a complete list of tested cytokines.

Beads were added to 96-well plates and washed in a Biotek ELx405 washer (Biotek Instruments Inc., Winooski, Vermont, USA). Sera samples were added to plates containing mixed antibody-linked beads and incubated at room temperature for 1 h. Those samples were incubated overnight at 4 °C with shaking. Cold and room temperature incubation steps were performed on an orbital shaker at 500–600 rotations per minute. Between steps, plates were washed. Following overnight incubation, biotinylated-detection antibodies were added to plates for 75 min at room temperature, while shaking. Plates were incubated at room temperature with streptavidin-PE (eBiosciences/Affymetrix, San Diego, California, USA) for 30 min. A reading buffer was added to wells on each plate. Each sample was measured in duplicate. Plates were read using a Luminex 200 instrument (Luminex Corporation, Austin, Texas, USA) with a lower bound of 50 beads per sample per cytokine. The assay measures cytokine concentration and reports results in units of median fluorescence intensity (MFI). Four custom assay control beads (Radix Biosolutions, Georgetown, Texas, USA) were added to all wells.

### Statistical methods

#### Linear models of titer and cytokines

We derived changes in RPR titer (log2) and cytokines by comparing the RPR titer value at time of incident syphilis to the RPR titer value taken from the prior visit. We then compared the linear model of RPR titer ~ infection (reduced model) to titer ~ infection + cytokine + infection × cytokine (full model), where titer and cytokine values are changes, deriving a p-value based on a partial F-test. Since this is a hypothesis generating study, we report unadjusted p-values. All statistical tests were performed in R (R, Vienna, Austria).

#### Comparison of changes in cytokine levels between “uninfected to infected” and “infected to uninfected” participant samples

To address the question of whether changes in cytokine concentrations are different when the patient is moving from an “uninfected to infected” as compared to “infected to treated,” we computed changes in cytokine concentrations (for example, a cytokine concentration was compared between incident syphilis and no syphilis). Samples were compared with other samples from an individual. We classified those changes as from “uninfected to infected” and from “infected to cured.” We compared those values using a t-test. Since this is a hypothesis generating study, we report unadjusted p-values.

#### Analysis of relationships between cytokine pairs

We considered all possible pairs of 63-cytokines (n = 1953) to identify relationships between pairs of cytokines. To do that, we divided values for each cytokine into 3 equal frequency tertiles or “bins” (low, medium, and high). Then, we focused on observations in low and high bins. For a particular pair of cytokines, we created a 2 × 2 array (cytokine 1, cytokine 2, low, high) and identified which 2 corners contained the most observations by comparing the number of observations in the lower left (low–low) and upper right (high–high) corners (positive correlation) to the number in the upper left (low–high) and lower right (high-low) (negative correlation). We next compared the number of observations associated among those with incident syphilis to those with no syphilis in the “left hand” and “right hand” corners. We calculated Chi squared values between those measures. We considered a p-value < 0.05 to be significant. We represented the significant relationships in a network graph using the R package, igraph. We then determined if those relationships could be used as classification rules, and identified which samples were correctly classified as “no infection” or “yes infection” by those rules. The resulting data were displayed in a heat map with hierarchical clustering (R method heatmap.2, with clustering method = complete). A similar analysis was used to identify which people were correctly classified by which clustering rules, with a correct classification for any sample considered to be a correct classification for each participant.

### Analysis of variability

To compare the variability of samples drawn from our current study in Lima, Peru to a control population, we identified 16 healthy males without syphilis between the ages of 20 to 40 years who participated in a flu vaccine study in Palo Alto, California [17]. Each of those control participants contributed 3 samples over a 30 days study period in either 1 or 2 years (3 or 6 total samples per person). All of those Palo Alto samples were analyzed on the same Luminex 63-plex kit (H51-2 and 12-10). Since the Peru cohort was analyzed on a different kit than control samples for age and sex, we also included control data from 4 healthy participants of a 14-week diet modification study (7 samples each) who were analyzed using the same Luminex kit as the Lima samples (H62-1 and 5-15). For each person and for each analyte, a percent coefficient of variation (%CV) was calculated as (standard deviation/mean) × 100. Differences between means of the 3 groups were tested using an ANOVA test without correction for multiple comparisons.

## Results

From a cohort of 401 participants, 44 participants with incident syphilis were identified. Among those 44 participants, 101 serum samples from different study visit dates (2 to 6 samples per participant depending on multiple cases of incident syphilis over the period of the study) were tested to assess serum cytokine concentrations. The median age of participants was 28.1 years (interquartile range 22.2, 31.8). Among the 44 participants with incident syphilis, 90.9% had a prior diagnosis of syphilis determined from serologic testing and 27.3% were co-infected with HIV. The groups without active syphilis had RPR titers that ranged from non-reactive to 1:8. RPR titres during incident syphilis ranged from 1:16 to 1:256. Cytokine concentrations and RPR titers varied at different time points. An example of longitudinal RPR titers and cytokine concentration is demonstrated with eotaxin (Additional file 1: Figure S1).

### Changes in cytokine concentration and RPR titer

**Fig. 1:**
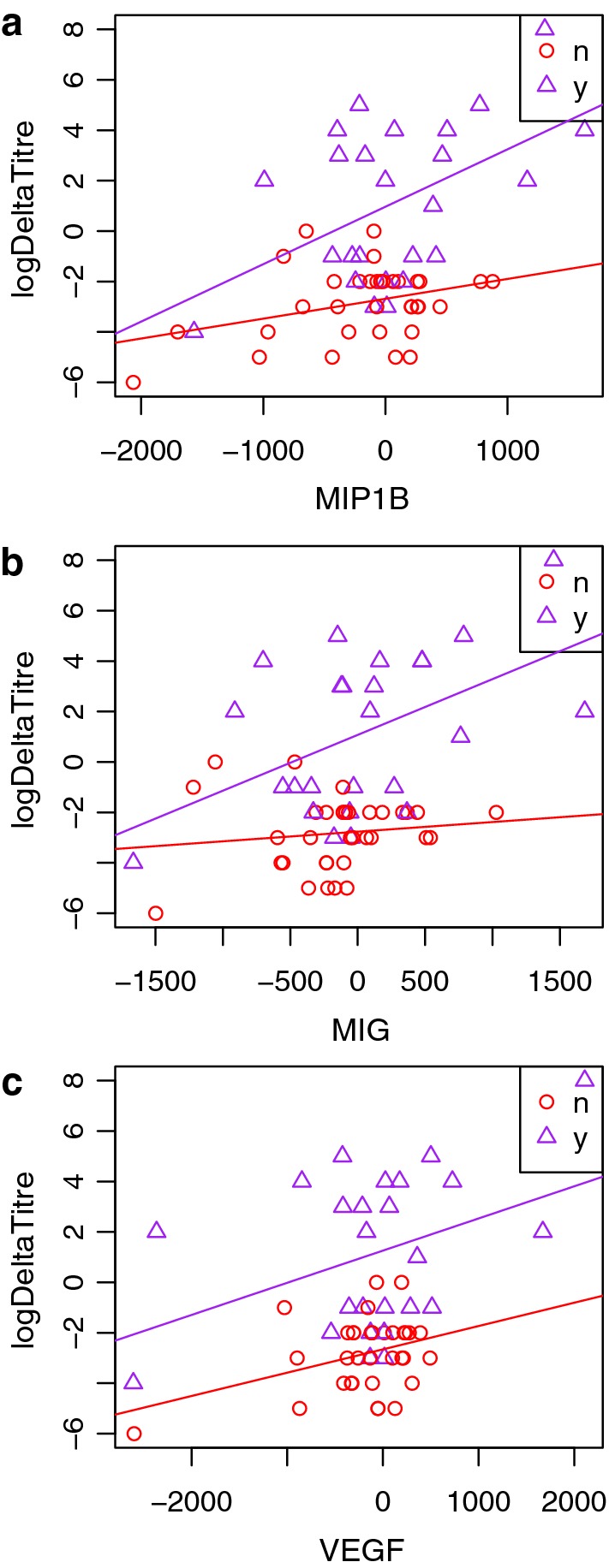
**a** Association between directionality of rapid plasma reagin titers and changes in cytokine concentration for MIP1B; **b** MIG; and **c** VEGF. Partial F test p-values were 0.001, 0.0026, and 0.011, respectively. Deltas in titer (log2) were derived as e.g. t1 − t0, with infection = y, n based on t1

Linear relationships between changes in RPR titer and cytokine concentrations were assessed among 40 participants at 59 time points. Among those participants, 29 participants had one point of comparison, 11 participants had two points, and one participant with five points. The three cytokines that were most highly associated with changes in RPR titer were MIP1B (p-value: 0.001, Fig. 1a), MIG (p-value: 0.0026, Fig. 1b), and VEGF (p-value: 0.011, Fig. 1c).

### Directionality of changes in RPR titer and cytokine concentrations

Statistically significant differences were found for Resistin (p-value: 0.014, Additional file 1: Figure S2a), BDNF (p-value: 0.026, Additional file 1: Figure S2b), IL23 (p-value: 0.026, Additional file 1: Figure S2c), Leptin (p-value: 0.023, Additional file 1: Figure S2d), Rantes (p-value: 0.022, Additional file 1: Figure S2e), and VCAM1 (p-value: 0.048, Additional file 1: Figure S2f).

### Predictive values of single cytokines to distinguish incident syphilis

Based on the two prior sets of results, none of the single cytokines could be used to reliably distinguish between samples from participants with active syphilis and treated infection.

### Pairwise cytokine analysis

**Fig. 2:**
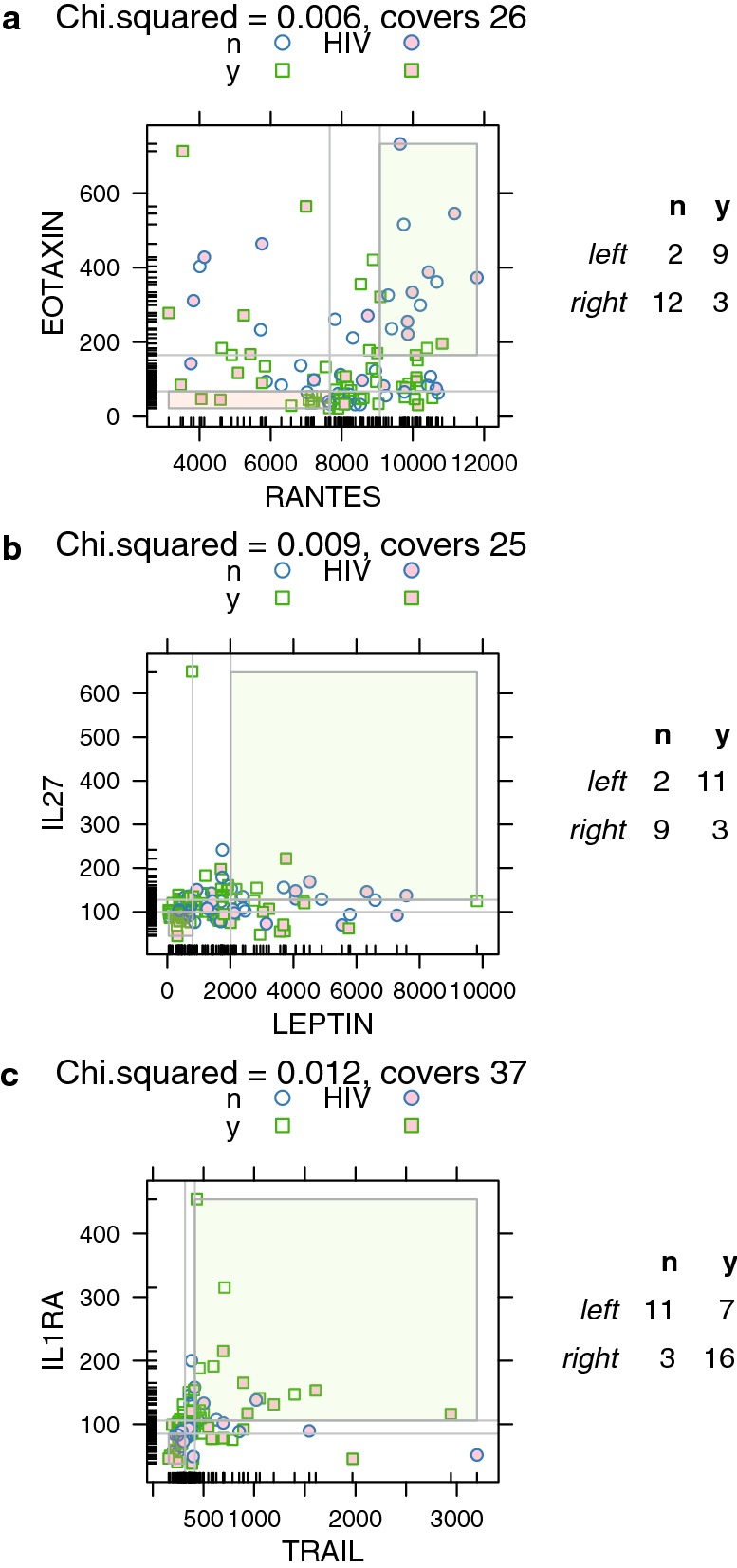
**a** Eotaxin and Rantes; **b** IL27 and Leptin; and **c** Il1RA and Trail. *The three pairs of cytokines that could best distinguish between people with incident syphilis versus people without syphilis. **a** The measurements for each cytokine were divided into tertiles, indicated by the light gray lines. Of the diagonally opposing pairs corners, RANTES low:EOTAXIN low (pink) and RANTES high:EOTAXIN high (green) contain more observations than did RANTES low:EOTAXIN high and RANTES high:EOTAXIN low. This lower left-hand corner (pink) contains 2 “no infection” samples and 9 “yes infection” samples. The right-hand corner (green) contains 12 “no infection” values and 3 “yes infection.” This difference is statistically significant, suggesting that samples with RANTES low and EOTAXIN low are more likely to suggest infection, while RANTES high and EOTAXIN high are more likely to suggest no infection. The title provides the Chi square p-value and number of observations in the highlighted corners corners. “No infection” samples are shown as circles while “yes infection” samples are shown as squares. Shading indicates samples from patients who are HIV+. The table on the right side of the graph shows counts of “no” and “yes” samples by corner. Black lines along the axes indicated observed values for that cytokine and represent the distribution of observed values. **b** Analysis as per A, for LEPTIN and IL27. **c** Analysis as per A, for TRAIL and ILRA

Paired cytokine analysis indicated differences in cytokine response between incident syphilis and no syphilis. Twenty pairs of cytokines (out of 1953 possible pairs) were identified that could be used to distinguish incident syphilis and cured syphilis (Chi squared test p-value < 0.05) in subsets of participants as described above in the methods section *Analysis of relationships between cytokine pairs*, and detailed in Table 1. Descriptions of the three pairs of cytokines that could best distinguish between incident syphilis and cured syphilis follows. Low Rantes and low Eotaxin were associated with incident syphilis whereas high Rantes and high Eotaxin were associated with treated infection (p-value: 0.006; odds ratio [OR], 18; 95% confidence interval [95% CI] 2.47, 131.29; Fig. 2a). Low Leptin and low IL27 were associated with incident syphilis whereas high Leptin and high IL27 were associated with treated infection (p-value: 0.009; OR, 16.5; 95% CI 2.25, 121.23; Fig. 2b). Low Trail and low IL1RA were associated with incident syphilis whereas high Trail and high IL1RA were associated with treated infection (p-value: 0.012; OR, 0.12; 95% CI 0.03, 0.57; Fig. 2c).

**Table 1:** Cytokine pairs classifying samples with incident syphilis

Cytokine 1	Cytokine 2	Chi squared p-value	Association	Covers	Left-no	Left-yes	Right-no	Right-yes	Rate-left	Rate-right	Rel risk	Odds-left	Odds-right	Odds ratio	95% CI
EOTAXIN	RANTES	0.006	Pos	26	2	9	12	3	0.82	0.20	4.09	4.50	0.25	18.00	2.47, 131.29
IL27	LEPTIN	0.009	Pos	25	2	11	9	3	0.85	0.25	3.38	5.50	0.33	16.50	2.25, 121.23
IL1RA	TRAIL	0.012	Pos	37	11	7	3	16	0.39	0.84	0.46	0.64	5.33	0.12	0.03, 0.57
IL1RA	MIG	0.028	Pos	35	11	6	4	14	0.35	0.78	0.45	0.55	3.50	0.16	0.04, 0.69
BDNF	MIG	0.028	Neg	25	10	3	3	9	0.23	0.75	0.31	0.30	3.00	0.10	0.02, 0.63
EOTAXIN	VCAM1	0.029	Pos	32	6	12	11	3	0.67	0.21	3.11	2.00	0.27	7.33	1.47, 36.67
FASL	LEPTIN	0.031	Pos	25	3	11	8	3	0.79	0.27	2.88	3.67	0.38	9.78	1.55, 61.65
LEPTIN	MCP1	0.034	Pos	23	3	12	6	2	0.80	0.25	3.20	4.00	0.33	12.00	1.56, 92.29
CD40L	IL22	0.037	Pos	25	7	8	0	10	0.53	1.00	0.53	1.13	21.00	0.05*	0.003, 1.09
EOTAXIN	VEGFD	0.038	Pos	23	1	9	8	5	0.90	0.38	2.34	9.00	0.63	14.40	1.38, 150.81
EOTAXIN	SCF	0.038	Pos	29	5	11	10	3	0.69	0.23	2.98	2.20	0.30	7.33	1.38. 38.88
LEPTIN	TNFB	0.038	Pos	29	5	11	10	3	0.69	0.23	2.98	2.20	0.30	7.33	1.38. 38.88
ENA78	MIG	0.038	Neg	24	10	2	4	8	0.17	0.67	0.25	0.20	2.00	0.10	0.01, 0.69
IL6	LEPTIN	0.040	Pos	26	5	10	9	2	0.67	0.18	3.67	2.00	0.22	9.00	1.39, 58.45
LEPTIN	RANTES	0.041	Pos	31	3	11	11	6	0.79	0.35	2.23	3.67	0.55	6.72	1.33, 33.91
IFNB	TRAIL	0.041	Pos	34	11	9	2	12	0.45	0.86	0.53	0.82	6.00	0.14	0.02, 0.78
EGF	MIG	0.041	Neg	29	12	5	3	9	0.29	0.75	0.39	0.42	3.00	0.14	0.03, 0.74
CD40L	TRAIL	0.044	Pos	26	8	5	2	11	0.38	0.85	0.45	0.63	5.50	0.11	0.02, 0.74
IFNG	IL1RA	0.045	Pos	36	9	8	3	16	0.47	0.84	0.56	0.89	5.33	0.17	0.04, 0.79
IL12P40	IL12P70	0.048	Pos	32	9	8	2	13	0.47	0.87	0.54	0.89	6.50	0.14	0.02, 0.80

* This table lists all pairs of cytokines for which the ratio of uninfected to infected samples is significantly different (Chi square p-value < 0.05) between the most common extreme tertiles [e.g., either low:low vs. high:high (positive association), or low:high vs high:low (negative association)]. The Chi square p-value is also provided, as well as the counts of uninfected (no) and infected (yes) samples in each corner. Corners are referred to as “Left” or “Right”, which generalizes to both positive and negative slopes. Pairs are ordered by p-value. The number of samples contained in the relevant extrema is shown in the “covers” column. This is the sum of the left-no, left-yes, right-no, and right-yes columns. Relative risk and odds ratios are also included. These calculations compare frequencies of incident syphilis in the left corner to those in the right corner. In some cases, the left corner has a higher frequency of incident syphilis than does the right corner, while in other cases the right corner has a higher frequency. These patterns are reflected in the relative risk and odds ratios. Odds ratio for CD40L-IL22 was computed using the Haldane–Anscombe correction to account for a zero in one cell

### Construction of cytokine networks

**Fig. 3:**
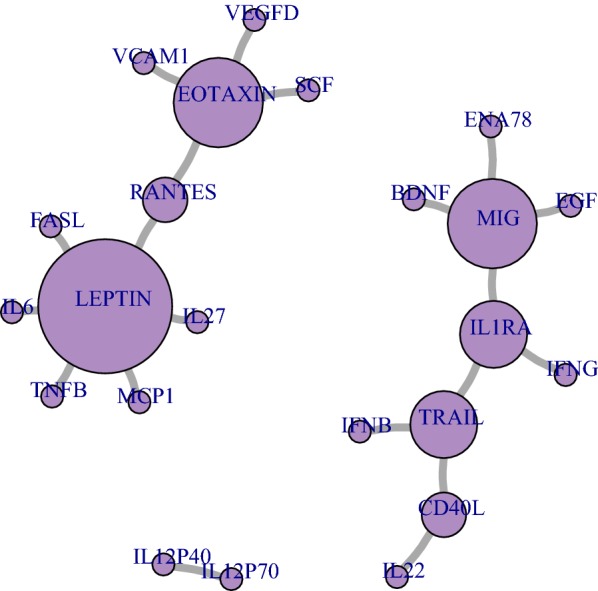
Cytokine networks identified from significant pairwise relationships among participants with incident syphilis. *Each circle represents a cytokine, and each line the relationship between them. Circle size is proportional to the number of connections

**Fig. 4:**
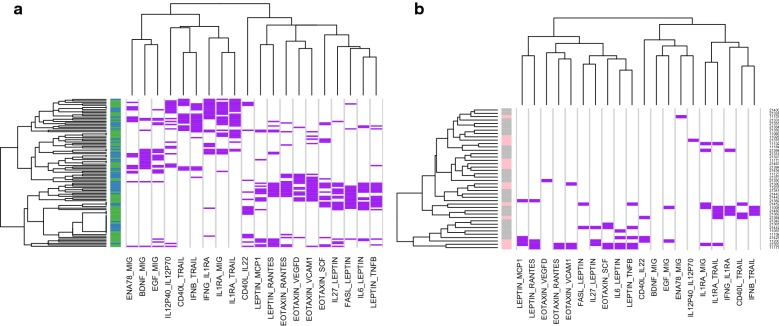
**a** Heatmap of hierarchical clustering of cytokine networks and participant samples with incident syphilis. Each row is a sample and each column is a cytokine pair. *Purple = correct classification. Blue shading indicates no syphilis while green shading indicates syphilis. **b** Heatmap of hierarchical clustering of cytokine networks and participants with and without human immunodeficient virus infection. Each row is a participant sample and each column is a cytokine pair. Purple squares indicate that the ratio associated with the cytokine pair correctly classified at least one sample from that participant. *Pink shading represents HIV-infected participants, while gray represents non HIV-infected participants

Three disjoint networks of interest formed in cytokine network construction: an Eotaxin–Rantes–Leptin network, a Mig-IL1ra-Trail-CD40L network, and an IL12p40-IL12p70 network (Fig. 3). Hierarchical clustering of participant samples by cytokine networks showed that participants’ infection state clustered into sub-groups by cytokine network (Fig. 4a). There was no difference between HIV-infected and HIV-uninfected participants’ infection state when hierarchical clustering rules were applied (Fig. 4b). Many cytokine pairs were highly sensitive and specific for the detection of incident syphilis (Additional file 1: Figure S3a). Application of six rules maximized cytokine pair sensitivity and specificity (Additional file 1: Figure S3b and c).

### Variability analysis

Eotaxin and MIG showed higher variability among samples collected from participants than control samples. Those data provide additional evidence of signal validity for the cytokines described in this study. The cytokines with no mean difference in %CV across the 3 cohorts are IL1B, TGFB, and IL13 (Additional file 1: Figure S4), illustrating that not all cytokines show increased variability in the study cohort.

## Discussion

This study explored the relationship between changes in cytokine concentrations and syphilis infection state by measuring 63 cytokines in serum samples from a longitudinal sample of MSM and transwomen from Lima, Peru. Though pairwise analysis, 20 cytokine pairs could distinguish serum samples collected from participants with incident syphilis and cured syphilis. We identified three cytokine networks of interest: an Eotaxin–Rantes–Leptin network, a Mig-IL1ra-Trail-CD40L network, and an IL12p40-IL12p70 network. We found that sub-groups of participants had different cytokine responses to incident syphilis: Some participants grouped into an Eotaxin–Rantes–Leptin network, whereas others grouped into a Mig-IL1ra-Trail-IL12p70 network. Finally, our analysis indicated there might be differences in cytokine concentration responses, particularly Resistin, BDNF, CD40L, and IFNB, during incident syphilis versus cured syphilis. While some participants were infected with HIV, there were no convincing clustering effects around HIV infection status. Comparisons between our cohort and control cohorts showed differences in variability by cytokine, with some cytokines showing higher variability in the study cohort. High cytokine variability is suggestive that differences in cytokine concentrations observed in this study between active syphilis and cured syphilis are likely true phenomena versus random variation.

We were unable to find similar studies for a comparison of our findings. Our current study design greatly differs from our previous smaller cross-sectional pilot study, which compared cytokine values from samples collected from 5 patients with syphilis (in which the duration of syphilis was unknown) to samples collected from 5 patients without syphilis [18]. While there were large methodological differences between those studies, our current study also found significant differences in Eotaxin, Leptin, IL12P70, and VEGF-D, which replicated some of the findings of our earlier study. We did not replicate findings of differences in GMCSF, IL10, IL15, IL1B, IL7, IP10, MCP3, MIP1B, NGF, PDGFBB, TNFA, and VEGF.

In addition to its role as an anorexigenic, leptin has also been found to be a proinflammatory factor [19]. In acute inflammation and infection, leptin levels increase due to the presence of bacterial endotoxins and other signaling cytokines like TNF-a, IL-6, and IL-1b [20, 21]. In experiments, *Klebsiella* spp. pulmonary infections are more lethal in leptin-deficient mice when compared to wild-type mice. It was also found that leptin administration into leptin-deficient mice reduced bacteriemia, improved macrophage phagocytosis, improved polymorphonuclear leukocyte H_2_O_2_ production, reduced bacterial load in vitro, and improved survival [22–26]. Leptin could have an important role in the immune response to syphilis, however it may also be elevated among our participants due to the treatment of syphilis with penicillin. A study among patients with the Jarisch-Herxheimer Reaction found that TNF, IL-6, and IL-8 were elevated 2 to 4 h after receiving penicillin treatment, with a return toward baseline values 12 h after receiving penicillin [27]. Therefore observed elevations in inflammatory cytokines could also be observed due to the treatment of syphilis versus incident syphilis.

There were several limitations to our study. Our analysis is based on data collected from a medium sized cohort (103 samples from 44 persons) characterized by confounders such as prior syphilis. Detection of changes in cytokines were limited to 3-month intervals due to the parent study design. There may be differences in cytokine expression among patients infected with HIV infection [28], however statistical analysis did not find differences between participants infected with HIV and participants not infected with HIV. There is variation in our data, as shown in Fig. 1. We were unable to determine if that variation was related to individual variability or to unidentified technical considerations. However, we performed a variability analysis that showed consistency in datasets when we compared our results with those from cohorts of matched healthy controls. There are several limitations in our identification of ratios for classifying samples. Our analysis did not consider correlation within multiple samples from the same person. In addition, the definition of tertiles was created from our data. Additional studies are needed to validate our identified rules and to derive general threshold values for low and high cytokine concentrations.

Our study has many strengths. In our study, we tested stored sera collected at regular intervals from a longitudinal cohort that allowed us to compare samples collected during incident syphilis to those collected 3 months prior. This allowed each participant to act as their own control. While prior studies have compared cytokine levels during an incident syphilis to those after treatment, we were able to compare cytokine levels prior to incident syphilis to those during incident syphilis. With that approach, we may have a better comparison group that is not influenced by variation between individuals. Additionally, quarterly testing allowed us to have a documented time frame in which incident syphilis took place. We performed an analysis using RPR titers as a continuous variable. Another strength of our analysis of ratios is that we identified pairs of cytokines that have the potential to distinguish a patient who does not need treatment for active syphilis from a patient who does. Those values can be determined in a single visit, unlike changes in cytokine concentrations over time. Future research is needed to confirm the presence of the cytokine networks we identified.

## Conclusions

Differences in cytokine profiles are present among MSM and transwomen with no syphilis, incident syphilis, and treated infection. Cytokine sets could be used to distinguish between samples with active infection versus none. Cytokine assays may be a potentially useful tool for aiding in the interpretation of the underlying biological activity of a reactive RPR titer. While our results need to be further replicated, there are many promising findings that could be useful in the development of new and better tests for the diagnosis and management of syphilis.

## Additional file

**Additional file 1.** Additional tables and figures.

## Data Availability

The datasets used and/or analyzed during the current study are available from the corresponding author on reasonable request.

## Abbreviations

RPR: rapid plasma reagin
MSM: men who have sex with men
TPPA: *Treponema pallidum* particle agglutination
HIV: human immunodeficiency virus
MFI: median fluorescence intensity

## References

[1] World Health Organization. Proposing 2016–2021 global health sector strategies for HIV, viral hepatitis and sexually transmitted infections (STIs). Briefing note: 16.09.2015: World Health Organization; 2015.

[2] Kojima N, Klausner JD (2018). An update on the global epidemiology of syphilis. Curr Epidemiol Rep..

[3] Workowski KA, Bolan GA, Centers for Disease C, Prevention (2015). Sexually transmitted diseases treatment guidelines, 2015. MMWR Recomm Rep..

[4] Morshed MG, Singh AE (2015). Recent trends in the serologic diagnosis of syphilis. Clin Vaccine Immunol.

[5] World Health Organization (2016). WHO guidelines for the treatment of *Treponema pallidum* (syphilis).

[6] Johnson PD, Graves SR, Stewart L, Warren R, Dwyer B, Lucas CR (1991). Specific syphilis serological tests may become negative in HIV infection. AIDS..

[7] Larsen SA, Steiner BM, Rudolph AH (1995). Laboratory diagnosis and interpretation of tests for syphilis. Clin Microbiol Rev.

[8] Clement ME, Okeke NL, Hicks CB (2014). Treatment of syphilis: a systematic review. JAMA.

[9] Zetola NM, Klausner JD (2007). Syphilis and HIV infection: an update. Clin Infect Dis.

[10] Ratnam S (2005). The laboratory diagnosis of syphilis. Can J Infect Dis Med Microbiol..

[11] Augenbraun M, French A, Glesby M, Sanchez-Keeland L, Young M, Greenblatt R (2010). Hepatitis C virus infection and biological false-positive syphilis tests. Sex Transm Infect..

[12] Miller JN (1975). Value and limitations of nontreponemal and treponemal tests in the laboratory diagnosis of syphilis. Clin Obstet Gynecol.

[13] Pastuszczak M, Gozdzialska A, Jakiela B, Obtulowicz A, Jaskiewicz J, Wojas-Pelc A (2017). Robust pro-inflammatory immune response is associated with serological cure in patients with syphilis: an observational study. Sex Transm Infect..

[14] Zhang RL, Wang QQ, Zhang JP, Yang LJ (2017). Molecular subtyping of *Treponema pallidum* and associated factors of serofast status in early syphilis patients: identified novel genotype and cytokine marker. PLoS ONE.

[15] Deiss RG, Leon SR, Konda KA, Brown B, Segura ER, Galea JT (2013). Characterizing the syphilis epidemic among men who have sex with men in Lima, Peru to identify new treatment and control strategies. BMC Infect Dis.

[16] Kojima N, Park H, Konda KA, Joseph Davey DL, Bristow CC, Brown B (2017). The PICASSO Cohort: baseline characteristics of a cohort of men who have sex with men and male-to-female transgender women at high risk for syphilis infection in Lima, Peru. BMC Infect Dis.

[17] Shen-Orr SS, Furman D, Kidd BA, Hadad F, Lovelace P, Huang YW (2016). Defective signaling in the JAK-STAT pathway tracks with chronic inflammation and cardiovascular risk in aging humans. Cell Syst..

[18] Kojima N, Bristow CC, Maecker H, Rosenberg-Hasson Y, Leon SR, Vargas SK (2016). Similarities in the markers of inflammation between men with syphilis and women with increased risk of HIV acquisition. Clin Infect Dis.

[19] Abella V, Scotece M, Conde J, Pino J, Gonzalez-Gay MA, Gomez-Reino JJ (2017). Leptin in the interplay of inflammation, metabolism and immune system disorders. Nat Rev Rheumatol.

[20] La Cava A (2017). Leptin in inflammation and autoimmunity. Cytokine.

[21] Sarraf P, Frederich RC, Turner EM, Ma G, Jaskowiak NT, Rivet DJ (1997). Multiple cytokines and acute inflammation raise mouse leptin levels: potential role in inflammatory anorexia. J Exp Med.

[22] Faggioni R, Fantuzzi G, Gabay C, Moser A, Dinarello CA, Feingold KR (1999). Leptin deficiency enhances sensitivity to endotoxin-induced lethality. Am J Physiol.

[23] Faggioni R, Moser A, Feingold KR, Grunfeld C (2000). Reduced leptin levels in starvation increase susceptibility to endotoxic shock. Am J Pathol.

[24] Hsu A, Aronoff DM, Phipps J, Goel D, Mancuso P (2007). Leptin improves pulmonary bacterial clearance and survival in ob/ob mice during pneumococcal pneumonia. Clin Exp Immunol.

[25] Wieland CW, Stegenga ME, Florquin S, Fantuzzi G, van der Poll T (2006). Leptin and host defense against Gram-positive and Gram-negative pneumonia in mice. Shock..

[26] Xiao E, Xia-Zhang L, Vulliemoz NR, Ferin M, Wardlaw SL (2003). Leptin modulates inflammatory cytokine and neuroendocrine responses to endotoxin in the primate. Endocrinology.

[27] Negussie Y, Remick DG, DeForge LE, Kunkel SL, Eynon A, Griffin GE (1992). Detection of plasma tumor necrosis factor, interleukins 6, and 8 during the Jarisch–Herxheimer Reaction of relapsing fever. J Exp Med.

[28] Kenyon C, Osbak KK, Crucitti T, Kestens L (2017). The immunological response to syphilis differs by HIV status; a prospective observational cohort study. BMC Infect Dis.

