# Supplementary material for: Cytokine expression in Treponema pallidum infection
Source: J Transl Med. 2019 Jun 11;17:196. doi: 10.1186/s12967-019-1947-7 (PMC6558693; doi:10.1186/s12967-019-1947-7)
Supplement: Supplementary file 1 — Additional file 1. Additional tables and figures. [file 12967_2019_1947_MOESM1_ESM.docx]

Table S1. List of cytokines tested by a multiplex bead-based enzyme-linked immunosorbent assay

| **Cytokines** | **Common names** |
| --- | --- |
| BDNF | Brain derived neurotrophic factor |
| CCL2 | C-C motif chemokine ligand 2 |
| CCL3 | C-C motif chemokine ligand 3 |
| CCL4 | C-C motif chemokine ligand 4 |
| CCL5 | C-C motif chemokine ligand 5 |
| CCL7 | C-C motif chemokine ligand 7 |
| CCL11 | C-C motif chemokine ligand 11 |
| CD40LG | CD40 ligand |
| CSF1 | Colony stimulating factor 1 |
| CSF2 | Colony stimulating factor 2 |
| CSF3 | Colony stimulating factor 3 |
| CXCL1 | C-X-C motif chemokine ligand 1 |
| CXCL10 | C-X-C motif chemokine ligand 10 |
| CXCL5 | C-X-C motif chemokine ligand 5 |
| CXCL9 | C-X-C motif chemokine ligand 9 |
| CXCL12 | C-X-C motif chemokine ligand 12 |
| EGF | Epidermal growth factor |
| FASLG | Fas ligand |
| FGF2 | Fibroblast growth factor 2 |
| HGF | Hepatocyte growth factor |
| ICAM1 | Intercellular adhesion molecule 1 |
| IFNA2 | Interferon alpha 2 |
| IFNB1 | Interferon beta 1 |
| IFNG | Interferon gamma |
| IL1A | Interleukin 1 alpha |
| IL1B | Interleukin 1 beta |
| IL1RA | Interleukin 1 receptor antagonist |
| IL2 | Interleukin 2 |
| IL4 | Interleukin 4 |
| IL5 | Interleukin 5 |
| IL6 | Interleukin 6 |
| IL7 | Interleukin 7 |
| IL8 | Interleukin 8 |
| IL9 | Interleukin 9 |
| IL10 | Interleukin 10 |
| IL12P70 | Interleukin 12 active heterodimer |
| IL12P40 | Interleukin 12 subunit beta |
| IL13 | Interleukin 13 |
| IL15 | Interleukin 15 |
| IL17A | Interleukin 17A |
| IL17F | Interleukin 17F |
| IL18 | Interleukin 18 |
| IL21 | Interleukin 21 |
| IL22 | Interleukin 22 |
| IL23 | Interleukin 23 |
| IL27 | Interleukin 27 |
| IL31 | Interleukin 31 |
| KITLG | KIT ligand |
| LEPTIN | Leptin |
| LIF | Interleukin 6 family cytokine |
| LTA | Lymphotoxin alpha |
| NGF | Nerve growth factor |
| PDGFBB | Platelet-derived growth factor subunit BB |
| RESISTIN | Resistin |
| SERPINE1 | Serpin family E member 1 |
| TGFA | Transforming growth factor alpha |
| TGFB | Transforming growth factor beta |
| TNFA | Tumor necrosis factor alpha |
| TNFSF10 | TNF superfamily member 10 |
| VCAM1 | Vascular cell adhesion molecule 1 |
| VEGF | Vascular endothelial growth factor |
| VEGFD | Vascular endothelial growth factor D |

Figure S1. Participant rapid plasma reagin titers and cytokine data from multiple visits.

Figure S2a. Differences in cytokine concentration between participants with incident syphilis and those without syphilis, Resistin

Figure S2b. BDNF

Figure S2c. IL23

Figure S2d. Leptin

Figure S2e. Rantes

Figure S2f. VCAM1

Figure S3a. Sensitivity and specificity by pairs of cytokines

Sensitivity and specificity are calculated independently for each cytokine pair. Letters on the plot correspond to letters in the adjacent table. Rules were sorted by descending order by sensitivity and sequentially applied to all samples. Once a sample was covered by a rule, it was removed from consideration for subsequent rules. The gray line illustrates cumulative sensitivity, while the pink line illustrates cumulative specificity.

Figure S3b. Sensitivity and specificity by pairs of cytokines

After application of the first 6 rules, sensitivity is 78% and specificity 65%, with 70 of 101 samples covered. Sensitivity decreases thereafter.

Figure S3c. Sensitivity and specificity by pairs of cytokines

Sorting rules by specificity yields a specificity plateau at about the 6^th^ rule, with sensitivity is 67% and specificity 82%, with 79 of 101 samples covered. The maximum sensitivity of 69% is reached by the 10^th^ rule.

Figure S4. Three cytokines with the least evidence for differing coefficient of variation values by cohort, as defined by largest p-values. This illustrates that not all cytokines are more variable in the syphilis cohort than in the healthy controls.
